# Medicaid managed care organization service coverage and diagnosis and treatment of opioid use disorder: evidence from quasi-random auto-assignment in Kentucky

**DOI:** 10.1093/haschl/qxaf119

**Published:** 2025-06-11

**Authors:** Shelby R Steuart, Miguel Antonio G Estrada, Christina M Andrews, Colleen M Grogan, Olivia M Hinds, Emily C Lawler, Lauren A Peterson, Felipe Lozano-Rojas, Melissa A Westlake, Coady Wing, Amanda J Abraham

**Affiliations:** Crown Family School of Social Work, Policy, and Practice, The University of Chicago, Chicago, IL 60637, United States; Department of Public Administration and Policy, University of Georgia, Athens, GA 30602, United States; Department of Health Services Policy and Management, Arnold School of Public Health, University of South Carolina, Columbia, SC 29208, United States; Crown Family School of Social Work, Policy, and Practice, The University of Chicago, Chicago, IL 60637, United States; Center for Health Administration Studies, The University of Chicago, Chicago, IL 60637, United States; Department of Health Services Policy and Management, Arnold School of Public Health, University of South Carolina, Columbia, SC 29208, United States; Department of Public Administration and Policy, University of Georgia, Athens, GA 30602, United States; National Bureau of Economic Research, Boston, MA 02138, United States; Crown Family School of Social Work, Policy, and Practice, The University of Chicago, Chicago, IL 60637, United States; Department of Public Administration and Policy, University of Georgia, Athens, GA 30602, United States; Department of Health Services Policy and Management, Arnold School of Public Health, University of South Carolina, Columbia, SC 29208, United States; School of Public and Environmental Affairs, Indiana University, Bloomington, IN 47405, United States; Department of Public Administration and Policy, University of Georgia, Athens, GA 30602, United States

**Keywords:** medicaid, medicaid managed care, opioid use disorder

## Abstract

**Introduction:**

Opioid-related mortality continues to claim tens of thousands of American lives annually. Medicaid plays an outsized role in financing opioid use disorder (OUD) treatment, paying for almost 40% of all Americans who received OUD treatment in 2017.

**Methods:**

Using Medicaid T-MSIS Analytic Files data and a novel data set of Medicaid managed care organization (MCO) plan coverage, we examined the relationship between comprehensiveness of benefits for OUD treatment provided by Medicaid MCO plans and the likelihood of OUD diagnosis and medications for OUD (MOUD) receipt among newly enrolled Medicaid beneficiaries in Kentucky. We use two stage least squares to adjust for MCO plan choice that may be correlated with individual OUD risk or individual demand for OUD treatment.

**Results:**

Our findings show that Medicaid beneficiaries assigned to MCO plans with more comprehensive OUD benefits are more likely to be diagnosed with OUD and to receive MOUD.

**Conclusion:**

These results suggest that increasing Medicaid MCO plan coverage to include a broader array of OUD treatment services and medications may be an effective strategy for increasing both OUD diagnosis and MOUD receipt, which is crucial for combating the ongoing opioid epidemic.

## Introduction

Despite substantial investment at state and federal levels aimed at curbing the opioid epidemic, opioid-related mortality continues to claim thousands of American lives annually.^1^ In 2022, a recorded 6.5 million Americans had an opioid use disorder (OUD), and over 81 000 died of an opioid-related overdose.^2^ Expanding receipt of medications for OUD (MOUD)^2^ has the potential to improve OUD treatment outcomes and reduce opioid-related mortality. However, in 2022, only 25% of those diagnosed with OUD received any MOUD.^3^

Medicaid plays an outsized role in the financing of OUD treatment, serving as payer for almost 40% of all Americans who received OUD treatment in 2017.^4^ Three-quarters of all Medicaid beneficiaries are enrolled in Medicaid managed care plans,^5^ suggesting that the design of Medicaid managed care plans and their approach to coverage of OUD treatment may play a central role in the response of state governments to the epidemic. Yet, Medicaid managed care organizations (MCOs) have considerable discretion over the services and medications they elect to reimburse, and the conditions under which services and medications are eligible for reimbursement,^6^ even when states have coverage mandates for MCOs.^7^ As a result, there is substantial variation in Medicaid MCO coverage of OUD treatment, and little is known regarding how MCO plan policies designed to monitor and limit OUD treatment affect OUD diagnosis and receipt of MOUD.^8^

One way to characterize the comprehensiveness of an MCO plan is the enumerate of the collection of services and medications used to diagnose and treat beneficiaries with OUD. In theory, restrictive coverage that excludes certain services may disrupt a chain of events that ultimately leads to diagnosis and treatment of OUD. Thus, it is plausible that Medicaid MCO plans that provide coverage for a broader array of “entry points” into specialty treatment could lead to more and faster diagnoses of OUD and higher rates of MOUD initiation. However, no studies to date have rigorously assessed the role of comprehensive OUD treatment coverage in facilitating OUD diagnosis and receipt of MOUD.

To address this gap, we focus on Kentucky's Medicaid program, in which beneficiaries are quasi-randomly assigned into 1 of 5 Medicaid MCO plans. Quasi-random assignment implies that the people assigned to each plan have comparable health and OUD risks at baseline. By exploiting quasi-random assignment, our study is the first to measure the relationship between the comprehensiveness of MCO plan coverage of OUD treatment and 2 outcomes: diagnosis of OUD and subsequent initiation of MOUD. We test the hypothesis that MCO plans with more comprehensive OUD treatment coverage (ie, plans facilitating access to OUD treatment through multiple “entry points”) will increase the probability of OUD diagnosis and treatment with MOUD. Understanding the role of MCO plan design on these outcomes has important public policy relevance because states can and often do impose specific requirements on the design of Medicaid MCO plans. If comprehensive OUD coverage is an effective tool for mitigating harms of the opioid epidemic, states may choose to make comprehensive coverage nondiscretionary.

## Study data and methods

### Data and study sample

Patient-level data come from the 2016-2018 Kentucky T-MSIS Analytic Files (TAF). We selected Kentucky because the state enrolled all beneficiaries into MCO plans during our study period and assigned beneficiaries to plans through an auto-assignment algorithm prior to beneficiary choice. These enrollment procedures enable a research design that exploits quasi-random assignment and has high internal validity. Additionally, Kentucky experienced high opioid mortality over the study period^9^ and possessed a relatively simple managed care arrangement with 5 MCO plans across 9 regions.

During the study period, Kentucky Medicaid auto-assigned new beneficiaries to 5 MCO plans. All MCO plans were available in all counties during the study period. Beneficiaries were allowed to change plans for 90 days after enrollment. Using month-by-month managed care enrollment in the TAF Medicaid Managed Care Supplement file, we categorized a beneficiary's first observed plan enrollment as their “assigned plan” and their final observed plan within the calendar year (whether it changes or not) as their “realized plan.” Over 95% of beneficiaries in our sample remained in their auto-assigned plan through the end of their second year of continuous enrollment, meaning only 5% of beneficiaries actively chose to switch plans (see Appendix Table S1).

Our study population consisted of 252 791 people newly enrolled in Medicaid in Kentucky. We measured several baseline covariates: sex (male, female), household size, age (18-33, 34-49, 50-64), and race/ethnicity (Hispanic/Latinx, Black, White, other/unknown race).

Our key independent variable was comprehensiveness of MCO plan coverage of OUD treatment, measured as the total number of OUD treatment services and medications covered in each plan-year (Table 1). Data on Medicaid MCO plan OUD treatment benefits were collected from publicly available plan documentation (member handbooks, provider manuals, and prescription drug formularies) for the 5 active MCO plans in Kentucky from 2016 to 2018 (Aetna Better Health of Kentucky, Anthem, Humana CareSource, Passport Health Plan, and WellCare of Kentucky). For each plan, we assessed whether there was specified coverage for a series of OUD treatment services (outpatient, intensive outpatient/partial hospitalization, outpatient or inpatient detoxification, residential, inpatient hospitalization, and recovery support services) and U.S. Food and Drug Administration-approved medications (methadone, injectable naltrexone, and buprenorphine), widely endorsed by the American Society of Addiction Medicine (ASAM).^10^

**Table 1.** Covered meds and services (comprising the comprehensiveness measure).

	Aetna	Anthem	Humana	Passport	WellCare
2016	Meds.: 1 Services: 3 Total: 4 NC: 2 NS: 2	Meds.: 1 Services: 5 Total: 6 NC: 1 NS: 1	Meds.: 2 Services: 4 Total: 6 NC: 0 NS: 2	Meds.: 2 Services: 6 Total: 8 NC: 0 NS: 0	Meds.: 1 Services: 6 Total: 7 NC: 1 NS: 1
2017	Meds.: 1 Services: 1 Total: 2 NC: 2 NS: 4	Meds.: 1 Services: 3 Total: 4 NC: 1 NS: 3	Meds.: 2 Services: 4 Total: 6 NC: 0 NS: 2	Meds.: 2 Services: 5 Total: 7 NC: 0 NS: 1	Meds.: 1 Services: 2 Total: 3 NC: 1 NS: 4
2018	Meds.: 1 Services: 3 Total: 4 NC: 2 NS: 2	Meds.: 1 Services: 2 Total: 3 NC: 1 NS: 4	Meds.: 2 Services: 3 Total: 5 NC: 0 NS: 3	Meds.: 2 Services: 6 Total: 8 NC: 0 NS: 0	Meds.: 1 Services: 4 Total: 5 NC: 1 NS: 2

Source: Authors’ analysis of primary data (MCO Coverage Data by State-Plan, collected by authors at the University of South Carolina). In the analysis, we code “not specified” as “no.” No plans cover methadone during this period and all plans covered buprenorphine.

Abbreviations: NC, not covered, NS, not specified.

We coded services that were not specified as not covered (not specified is abbreviated as NS in Table 1; not covered is abbreviated as NC in Table 1). For OUD treatment services, in 2016, 17% of possibly covered services (across all plans) were not specified; in 2017, 47% were not specified; and in 2018, 37% of possible services were not specified. For medications, buprenorphine was covered by all MCO plans for the entire study period, methadone was not specified by any plans for the entire study period, and injectable naltrexone was covered by 2 MCO plans for the entire study period and not covered by the other MCO plans for the entire study period (Table 1). In terms of coverage of OUD treatment services and MOUD, the services that were covered the least frequently were injectable naltrexone and detoxification. Injectable naltrexone was most frequently not covered and detoxification was most frequently not specified. Buprenorphine was always covered during our study period, and outpatient services were covered in all but 2 plan-years, making these the 2 most frequently covered OUD-related treatments.

The MCO comprehensiveness measure was standardized so that the lowest overall value of plan coverage was equal to 0. Given that no MCO plans covered methadone during the study period and that buprenorphine was covered in all plan-years during the study period, our measure of plan comprehensiveness primarily captures differences in coverage of OUD treatment services across plan-years.

### Study variables

We identified OUD diagnoses using ICD-10-CM codes from the Centers for Medicaid and Medicare Services (CMS), following the Chronic Conditions Warehouse algorithm.^11,12^ We identified MOUD using national drug codes (buprenorphine, injectable naltrexone) and HCPCS codes (buprenorphine, injectable naltrexone) from CMS.^11^ None of the 5 MCO plans in our study covered methadone during our study period, and we did not find evidence of methadone receipt in any Medicaid claims for beneficiaries.

Analyses included beneficiaries who were newly enrolled in Medicaid, aged 18-64, had at least three continuous months of Medicaid enrollment, and did not experience breaks in coverage of more than 1 month. We examined OUD diagnosis and receipt of MOUD among 2 groups: newly enrolled beneficiaries continuously enrolled for at least 3 months in calendar year 2016, 2017, or 2018 (*N* = 252 791) and newly enrolled beneficiaries continuously enrolled for at least 6 months across calendar years 2016-2017, or 2017-2018 (*N* = 165 936), with at least 3 months in each year (Appendix Table S2). In the first group, we constructed a dichotomous variable indicating if a beneficiary was diagnosed with OUD in the first calendar year of enrollment and a second dichotomous variable indicating if a beneficiary was diagnosed with OUD *and* received MOUD in the first calendar year of enrollment. In the second group, we constructed a dichotomous variable indicating if a beneficiary was diagnosed with OUD at any time during the 2 calendar years, and similarly, a second dichotomous variable indicating if a beneficiary was diagnosed with OUD *and* received MOUD at any point during the 2 calendar years.

### Statistical analysis

To provide evidence that MCO plan auto-assignment approximated randomization, we examined covariate balance on baseline (preenrollment) covariates across groups defined by auto-assigned plans. Appendix Tables S3-S6 report Cohen's D statistics for each baseline covariate for each assigned plan-year compared with the state average across all plans in that year, separately for urban and rural counties. As indicated by low Cohen's D statistics, the covariates were highly balanced across plans (Cohen’s D ≤ |0.15|), supporting the assumption that initial plan assignments were quasi-randomly assigned and not correlated with any preexisting health care risks or needs.^13^ The rates of plan switching we found were consistent with Marton et al.,^14^ who used Kentucky Medicaid administrative data for the plan that operated in our study period.

Because a small fraction of beneficiaries opted-out of their auto-assigned plan—a choice that may be correlated with individual OUD risk or individual demand for OUD treatment—we analyzed the data using both intent-to-treat (ITT) and instrumental variable (IV) estimators. Statistical analyses were conducted using Stata 18.0.

Our analysis distinguished between “assigned comprehensiveness” and “realized comprehensiveness.” A person's assigned comprehensiveness was defined as the comprehensiveness score of his/her auto-assigned MCO plan. Realized comprehensiveness was defined as the comprehensiveness of his/her realized plan. Thus, assigned and realized scores may differ if an enrollee opts out of the assigned plan.

In our ITT analysis, we regressed downstream outcomes (OUD diagnosis and MOUD treatment) on assigned plan comprehensiveness. In our IV analysis, we used a 2-stage approach. In the first stage, we regressed *realized* comprehensiveness on *assigned* comprehensiveness. In the second stage, to assess the impact of realized OUD comprehensiveness, we regressed downstream outcomes (OUD diagnosis and MOUD treatment) on predicted comprehensiveness from the first stage. We estimated both simple ITT and IV regressions as well as specifications that adjusted for county fixed effects to adjust for time-invariant unobserved confounders, year fixed effects to adjust for common trends across counties, and baseline covariates to adjust for any remaining compositional differences across counties. We used linear regressions in our main analysis.^15^ We obtained similar results in sign and magnitude using logistic regressions estimated using Two Stage Residual Inclusion (Appendix Table S7).^16^ We report our main specification results in a way consistent with previous *Health Affairs* publications conducted using 2-stage least squares^17^ and also include full tables in the Appendix that provide additional context (Appendix Tables S8 and S9).

We conducted subpopulation analyses evaluating the strength of our analyses in 2 high-risk populations (Appendix Tables S10 and S11). We conducted a supplementary analysis in which the comprehensiveness measure excluded MOUD and included only covered services (eg, IOP, residential, detox, recovery). Results from this specification remained statistically significant and similar in magnitude to the main results (Appendix Tables S12 and S13). Our results were also robust to the inclusion of 2 measures of buprenorphine provider availability (the ratio of unique, buprenorphine providers to patients per plan-county-year and the ratio of unique, buprenorphine providers with documented DEA X waivers to patients per plan-county-year) (Appendix Tables S14 and S15).

### Limitations

This study has several limitations. First, our study utilizes Kentucky TAF data which the Mathematica Data Quality (DQ) Atlas has noted may have issues in enrollment spans, enrollment numbers, and other services billing provider NPI numbers.^18^ However, we have been largely successful in developing strategies to overcome these challenges (see Appendix Table S16 for T-MSIS Analytic Files Analysis Reporting (TAR) Checklist). For example, while data on enrollment periods were thought to be unusable, we found high-quality enrollment periods using monthly managed care enrollment data from the Demographics and Eligibility (DE) Managed Care (MC) file (which the DQ Atlas note is of “low concern”).^18^

Second, there are many ways to quantify MCO plan comprehensiveness and utilization management is an important aspect of benefit design. However, given the large number of services and medications, and extensive array of utilization management policies, we elected to focus on a singular construct—OUD treatment coverage—to limit analytic complexity.

Third, in our analysis, we infer each person's “assigned plan” and “realized plan.” We do not have access to administrative data that would be needed to confirm plan assignments and opt outs. However, it is reasonable to infer because Kentucky Medicaid MCO contracts explicitly define the auto-assignment process as first assigning new beneficiaries to MCO plans and then providing them 90 days to change plans. Fourth, we abstracted data regarding MCO plan benefits from publicly available documentation provided by MCO plans. This documentation does not always explicitly note when a service or medication is covered. Finally, we only observe diagnoses/treatment for encounters that Medicaid covered and reimbursed.

Finally, it is possible that plans offering more comprehensive OUD coverage are also more progressive along other dimensions not accounted for in our analyses that may impact OUD diagnosis and MOUD initiation, such as measures of network breadth, network quality, and use of utilization management policies. While we were unable to measure network quality, we estimated models including 2 measures of network breadth, and these results were robust to the inclusion of these 2 measures (Appendix Tables S14 and S15). Additionally, we were unable to include measures of utilization management policies due to a lack of variation in the use of these policies (ie, only 1 plan in 1 year reported requiring prior authorization).

## Study results

### Sample characteristics

Demographic characteristics were similar in the first and second calendar year of enrollment (Table 2). In the first calendar year of enrollment, 3.7% of beneficiaries were diagnosed with OUD (SD: 0.19) and 1.5% of beneficiaries were diagnosed with OUD and received MOUD (SD: 0.12). In the second calendar year of enrollment, 5.8% of beneficiaries were diagnosed with OUD (SD: 0.23) and 3.4% of beneficiaries were diagnosed with OUD and received MOUD (SD: 0.18).

**Table 2.** Summary statistics.

	(2)		(3)	
	First calendar year		Second calendar year	
	Mean	SD	Mean	SD
Proportion female	0.506	0.500	0.503	0.500
Avg. household size	1.831	1.302	1.835	1.310
Proportion White	0.642	0.479	0.662	0.473
Proportion Hispanic/Latinx	0.036	0.185	0.031	0.175
Proportion Black/African American	0.107	0.310	0.107	0.310
Proportion missing race and ethnicity	0.198	0.399	0.183	0.387
Proportion age 18-33	0.475	0.499	0.470	0.499
Proportion age 34-49	0.316	0.465	0.320	0.466
Proportion age 50-64	0.209	0.407	0.210	0.407
Proportion diagnosed with OUD	0.037	0.189	0.058	0.234
Proportion diagnosed with OUD and filled MOUD	0.015	0.122	0.034	0.180
Observations	252 791		165 936	

Source: Authors’ analysis of secondary data (Medicaid TAF data). Beneficiaries with missing race and ethnicity variables are coded as “0” in each race and ethnicity category and “1” in the missing race category.

### Regression results

Table 3 reports estimates of the association between comprehensive OUD coverage and OUD diagnosis in the first calendar year (left panel) and second calendar year (right panel) samples. The 2-stage least squares results in Table 2 showed that being assigned to a plan with a 1-point higher comprehensiveness score increased the OUD diagnosis rate by 0.36% points in the first calendar year of enrollment and increased the OUD diagnosis rate by 0.55% points in the second calendar year. These results include county and year fixed effects and covariates. Relative to their baseline diagnosis rate, a 1-unit increase in OUD comprehensiveness increased the OUD diagnosis rate by 9.62% in the first calendar year of plan coverage and by 9.36% in the second calendar year.

**Table 3.** Plan comprehensiveness and OUD diagnosis.

	First calendar year			Second calendar year		
	OUD Dx (%)	Difference (percentage points)	Difference (%)	OUD Dx (%)	Difference (percentage points)	Difference (%)
Plan comprehensiveness	3.70	0.356***	9.62%	5.82	0.545***	9.36%
County fixed effects	X	X	X	X	X	X
Year fixed effects	X	X	X	X	X	X
Covariates	X	X	X	X	X	X

Source: Authors’ analysis of secondary data (Medicaid TAF data). The table presents estimates from a 2-stage least squares model. In the first stage, we regressed *realized* comprehensiveness on *assigned* comprehensiveness. In the second stage, to assess the impact of realized OUD comprehensiveness, we regressed downstream outcomes (OUD diagnosis) on predicted comprehensiveness from the first stage. All regression models controlled for sex, race and ethnicity, age, family size, and county and year fixed effects. *P < 0.10; **P < 0.05; ***P < 0.01.

Table 4 reports estimates of the association between plan comprehensiveness and the probability of receiving both an OUD diagnosis and MOUD. Results indicate that a 1-point higher comprehensiveness score led to a 0.14% point increase in the probability of OUD diagnosis and MOUD receipt in the first calendar year and a 0.27% point increase in OUD diagnosis and MOUD receipt in the second calendar year. These results are equivalent to an 8.95% increase in MOUD receipt in the first calendar year of plan coverage and an 8.03% increase in MOUD receipt in the second calendar year, over their respective baselines. These results include covariates and fixed effects.

**Table 4.** Plan comprehensiveness and OUD diagnosis + MOUD.

	First calendar year			Second calendar year		
	OUD Dx + MOUD receipt (%)	Difference (percentage points)	Difference (%)	OUD Dx + MOUD receipt (%)	Difference (percentage points)	Difference (%)
Plan comprehensiveness	1.52	0.136***	8.95%	3.40	0.273***	8.03%
County fixed effects	X	X	X	X	X	X
Year fixed effects	X	X	X	X	X	X
Covariates	X	X	X	X	X	X

Source: Authors’ analysis of secondary data (Medicaid TAF data). The table presents estimates from a 2-stage least squares model. In the first stage, we regressed *realized* comprehensiveness on *assigned* comprehensiveness. In the second stage, to assess the impact of realized OUD comprehensiveness, we regressed downstream outcomes (OUD diagnosis) on predicted comprehensiveness from the first stage. All regression models controlled for county and year fixed effects and the following covariates: sex, race and ethnicity, age, family size. *P < 0.10; **P < 0.05; ***P < 0.01.

## Discussion

Overall, we found that increases in Medicaid MCO plan comprehensiveness were associated with both increases in OUD diagnoses and MOUD receipt. Our estimates suggest that if all MCO plans in Kentucky had the same level of coverage as the plan with the most comprehensive coverage, while ensuring network adequacy, the OUD diagnosis rate of adults aged 18-64 would be 13.7% higher than it is currently. This is equivalent to diagnosing almost 9000 more adults with OUD in 2018. Our results indicate that comprehensive OUD coverage is an effective tool for mitigating the harm of the opioid epidemic; thus, it may be beneficial for states to make comprehensive coverage nondiscretionary.

Our results highlight the importance of providing Medicaid coverage for multiple points of entry into OUD treatment, which are often crucial for successful initiation of MOUD. In many parts of the country, OUD patients may be able to access specialty OUD treatment more easily than MOUD.^19,20^ Prior research on OUD treatment has focused largely on the receipt of MOUD, such as buprenorphine and methadone.^19-24^ Research unambiguously indicates MOUD, even without additional services, greatly reduces patients’ risk of overdose and death.^19-24^ However, our results provide support for the importance of OUD treatment services. While there was relatively little variation in medication coverage over the study period, coverage for services varied quite dramatically and thus was likely the key driver of the differences in OUD outcomes. Therefore, our findings are consistent with existing treatment models, such as the widely endorsed Opioid Cascade of Care, which identifies initiation and engagement in treatment services as an important gateway to subsequent medication initiation, often through repeated contact with specialty treatment providers and the establishment of a successful therapeutic alliance. Results are also consistent with ASAM guidance regarding the importance of comprehensive health plan coverage across the continuum of care for OUD.^10^

Finally, our study findings are consistent with prior work documenting substantial variability in Medicaid MCO plan benefits for OUD treatment. Our MCO coverage data set indicates that the most comprehensive plan-year covers 8 medications and services (buprenorphine, injectable naltrexone, outpatient, IOP, any detox, inpatient, residential, recovery services) while the least comprehensive plan-year covers only 2 (buprenorphine and inpatient). Auto-assigning Medicaid beneficiaries to a plan with only 2 covered forms of treatment when a plan with 8 is available to other beneficiaries raises significant questions regarding equity and ethical access to treatment. Given the ongoing epidemic of opioid-related overdose and mortality, these inequities can have deadly consequences.

## Conclusion

This study provides the first evidence that Medicaid beneficiaries enrolled in Medicaid MCO plans with more comprehensive coverage for OUD treatment have a higher probability of OUD diagnosis and receipt of MOUD, employing a study design that minimizes the challenges presented by selection bias that are widespread in studies evaluating the effects of health insurance design. These results highlight the importance of providing comprehensive OUD treatment service benefits in addition to coverage of MOUD to facilitate OUD diagnosis and receipt of MOUD. Our study points to the need for further research to understand how coverage of OUD treatment services acts as an engagement pathway to help Medicaid beneficiaries enter OUD treatment, receive a diagnosis, and make the decision to initiate MOUD.

## Supplementary Material

qxaf119_Supplementary_Data

## References

[1] Centers for Disease Control and Prevention . U.S. Overdose Deaths Decrease in 2023, First Time Since 2018 [Internet]. 2024. Cited August 2, 2024. Accessed July 15, 2024. https://www.cdc.gov/nchs/pressroom/nchs_press_releases/2024/20240515.htm

[2] Jones CM, Han B, Baldwin GT, Einstein EB, Compton WM. Use of medication for opioid use disorder among adults with past-year opioid use disorder in the US, 2021. JAMA Netw Open. 2023;6(8):e2327488. 10.1001/jamanetworkopen.2023.27488 37548979 PMC10407686

[3] Dowell D . Treatment for opioid use disorder: population estimates United States, 2022. MMWR Morb Mortal Wkly Rep [Internet]. 2024;73:567–574. https://www.cdc.gov/mmwr/volumes/73/wr/mm7325a1.htm 38935567 10.15585/mmwr.mm7325a1 PMC11254342

[4] Orgera K, Tolbert J. The Opioid Epidemic and Medicaid's Role in Facilitating Access to Treatment [Internet]. 2019. Cited June 10, 2024. Accessed June 9, 2024. https://www.kff.org/medicaid/issue-brief/the-opioid-epidemic-and-medicaids-role-in-facilitating-access-to-treatment/

[5] Kaiser Family Foundation . Total Medicaid MCO Enrollment [Internet]. 2024. Cited June 10, 2024. Accessed June 7, 2024. https://www.kff.org/other/state-indicator/total-medicaid-mco-enrollment/

[6] Peterson LA, Andrews CM, Abraham AJ, Westlake MA, Grogan CM. Most states allow medicaid managed care plans discretion to restrict substance use disorder treatment benefits. Health Affairs [Internet]. 2024;43:1038–1046. 10.1377/hlthaff.2023.01023 38950296

[7] Hinton E, Raphael J. 10 Things to Know About Medicaid Managed Care [Internet]. KFF; 2024. https://www.kff.org/medicaid/issue-brief/10-things-to-know-about-medicaid-managed-care/

[8] Medicaid and CHIP Payment and Access Commission . June 2019 Report to Congress on Medicaid and CHIP [Internet]. MACPAC. 2019. Cited June 10, 2024. Accessed June 9, 2024. https://www.macpac.gov/publication/june-2019-report-to-congress-on-medicaid-and-chip/

[9] Kentucky Justice & Public Safety Cabinet . 2020 Overdose Fatality Report [Internet]. 2021. Cited July 4, 2024. Accessed June 25, 2024. https://justice.ky.gov/news/pages/2020overdosereport.aspx.

[10] Crotty K, Freedman KI, Kampman KM. Executive summary of the focused update of the ASAM national practice guideline for the treatment of opioid use disorder. J Addict Med. 2020;14(2):99–112. 10.1097/ADM.0000000000000635 32209915

[11] MACBIS . CODE SETS FOR SUBSTANCE USE DISORDER (SUD) [Internet]. Cited June 28, 2024. Accessed June 20, 2024. https://www.medicaid.gov/medicaid/data-and-systems/downloads/macbis/sud_reference_codes.xlsx

[12] Chronic Conditions Data Warehouse . Other Chronic Health, Mental Health, and Potentially Disabling Conditions [Internet]. Cited 7 August, 2024. Accessed August 1, 2023. https://www2.ccwdata.org/web/guest/condition-categories-other

[13] Ho DE, Imai K, King G, Stuart EA. Matching as nonparametric preprocessing for reducing model dependence in parametric causal inference. *Polit Anal*. 2007;15(3):199-236. 10.1093/pan/mpl013.

[14] Marton J, Yelowitz A, Talbert JC. Medicaid program choice, inertia and adverse selection. J Health Econ. 2017;56:292–316. 10.1016/j.jhealeco.2017.04.006 29248057

[15] Abe K, Kawachi I, Watanabe T, Tamiya N. Association of the frequency of in-home care services utilization and the probability of in-home death. JAMA Netw Open. 2021;4(11):e2132787. 10.1001/jamanetworkopen.2021.32787 34748009 PMC8576578

[16] Terza JV . Two-stage residual inclusion estimation in health services research and health economics. Health Serv Res. 2018;53(3):1890–1899. 10.1111/1475-6773.12714 28568477 PMC5980262

[17] Lee D, Li J. Medicare enrollment increased visits to primary care providers but not mental health care providers, 2014–21. Health Affairs [Internet]. 2025;44(1):23–31. 10.1377/hlthaff.2024.00666 39761455

[18] Mathematica and Medicaid.gov . Welcome to the Medicaid Data Quality Atlas Internet. Cited August 28, 2024. Accessed November 1, 2023. https://www.medicaid.gov/dq-atlas/landing/states/single?state=21&tafVersionId=7

[19] Taylor JL, Samet JH. Opioid use disorder. Ann Intern Med [Internet]. 2022;175(1):ITC1–ITC16. 10.7326/AITC202201180 35007147

[20] Chan B, Gean E, Arkhipova-Jenkins I, et al Retention Strategies for Medications for Addiction Treatment in Adults With Opioid Use Disorder: A Rapid Evidence Review. Rockville (MD): Agency for Healthcare Research and Quality (US); 2020. 32775956

[21] Weiss RD, Potter JS, Fiellin DA, et al Adjunctive counseling during brief and extended buprenorphine-naloxone treatment for prescription opioid dependence: a 2-phase randomized controlled trial. Arch Gen Psychiatry. 2011;68(12):1238–1246. 10.1001/archgenpsychiatry.2011.121 22065255 PMC3470422

[22] Zoorob R, Kowalchuk A, Mejia de Grubb M. Buprenorphine therapy for opioid use disorder. Am Fam Physician. 2018;97(5):313–320. 29671504

[23] Shulman M, Wai JM, Nunes EV. Buprenorphine treatment for opioid use disorder: an overview. CNS Drugs. 2019;33(6):567–580. 10.1007/s40263-019-00637-z 31062259 PMC6585403

[24] Mariolis T, Bosse J, Martin SA, Wilson A, Chiodo LM. A systematic review of the effectiveness of buprenorphine for opioid use disorder compared to other treatments: implications for research and practice. J Addict Res Ther [Internet]. 2019. 10;2. 10.4172/2155-6105.1000379

