# Supplementary material for: Medicaid managed care organization service coverage and diagnosis and treatment of opioid use disorder: evidence from quasi-random auto-assignment in Kentucky
Source: Health Aff Sch. 2025 Jun 11;3(7):qxaf119. doi: 10.1093/haschl/qxaf119 (PMC12215311; doi:10.1093/haschl/qxaf119)
Supplement: qxaf119_Supplementary_Data [file qxaf119_supplementary_data.zip › Appendix_revised.docx]

**Medicaid MCO Service Coverage and Diagnosis and Treatment of Opioid Use Disorder**

Evidence from Quasi-Random Auto-Assignment in Kentucky

**APPENDIX**

Appendix Table 1: Assigned and Realized Plans: Second Calendar Year

|  | 2^nd^ Year Realized | | | | |  |
| --- | --- | --- | --- | --- | --- | --- |
| 1^st^ Year Assigned | Aetna | Anthem | Wellcare | Humana | Passport | Total |
| Aetna | 95% | 1% | 1% | 1% | 2% | 13,517 |
| Anthem | 1% | 94% | 2% | 1% | 2% | 39,524 |
| Wellcare | 0% | 1% | 98% | 1% | 1% | 41,206 |
| Humana | 0% | 1% | 2% | 95% | 2% | 29,523 |
| Passport | 0% | 1% | 2% | 1% | 96% | 42,166 |
| Total | 13,421 | 38,287 | 42,478 | 29,252 | 42,498 | 165,936 |

**​​**

Appendix Table 2: Sample Size by Year

| **Enrollment Year** | **One-year** | **Two-year** | **Attrition** |
| --- | --- | --- | --- |
| 2016 | 105,310 | 93,460 | 11,850 (11.3%) |
| 2017 | 85,286 | 72,476 | 12,810 (15%) |
| 2018 | 62,195 | None |  |
| Resulting Sample Size | 252,791 | 165,936 |  |

Note: “Attrition” describes individuals who leave Medicaid at the end of their first calendar year of Medicaid enrollment.

Individuals who change plans throughout the study period are not considered part of attrition counts, they remain in the sample.

*Balancing Tables (by Group and Urbanicity)*

Appendix Table 3: Cohen’s D - First Calendar Year- Two Urban Counties

|  | (1) | (2) | (3) | (4) | (5) |
| --- | --- | --- | --- | --- | --- |
|  | Aetna | Anthem | Humana | Passport | Wellcare |
| Age | 0.023 | 0.041 | 0.029 | -0.025 | -0.056 |
| % Female | -0.010 | 0.022 | 0.009 | -0.036 | 0.067 |
| Household Size | -0.005 | -0.077 | -0.064 | 0.071 | 0.001 |
| % Hispanic/Latinx | 0.015 | -0.120 | -0.053 | 0.045 | 0.096 |
| % Black | -0.040 | -0.065 | -0.027 | 0.076 | -0.059 |
| % AAPI | -0.006 | -0.021 | -0.000 | 0.014 | -0.003 |
| % Native American | -0.024 | 0.012 | 0.011 | -0.009 | -0.007 |
| % White | -0.066 | 0.107 | 0.000 | -0.027 | -0.060 |
| % Unknown Race | 0.099 | 0.013 | 0.056 | -0.080 | 0.058 |
| % Primary Language Spanish | 0.019 | -0.143 | -0.072 | 0.054 | 0.109 |
| % Primary Language English | -0.018 | 0.141 | 0.072 | -0.053 | -0.109 |
| % 0-100% FPL | -0.023 | -0.012 | -0.018 | 0.014 | 0.021 |
| % 101-133% FPL | -0.005 | 0.005 | 0.028 | -0.008 | -0.021 |
| % 134-150% FPL | 0.036 | 0.006 | -0.001 | -0.014 | 0.003 |
| % 151-200% FPL | 0.028 | 0.013 | -0.018 | -0.004 | -0.013 |
| % 201-255% FPL | 0.026 | 0.009 | -0.016 | -0.010 | -0.004 |
| % 256-300% FPL | -0.031 | 0.009 | -0.023 | -0.009 | 0.002 |
| % 301-400% FPL | -0.024 | -0.041 | -0.001 | 0.013 | -0.037 |
| % over 401% FPL | 0.013 | -0.025 | 0.002 | -0.010 | -0.025 |
| % Veterans | -0.006 | 0.017 | 0.028 | -0.005 | -0.074 |
| % US Citizens | 0.064 | 0.128 | 0.068 | -0.105 | 0.009 |
| Doctors attached to Any MOUD Claim | 0.569 | -0.885 | 1.207 | -0.611 | 1.125 |
| Doctors Providing Bup with Any DEA Bup Waiver | 0.344 | -1.016 | 1.503 | -0.504 | 0.801 |
| Observations | 5741 | 13901 | 11014 | 23934 | 8012 |

Appendix Table 4: Cohen’s D - First Calendar Year - All Other Counties

|  | (1) | (2) | (3) | (4) | (5) |
| --- | --- | --- | --- | --- | --- |
|  | Aetna | Anthem | Humana | Passport | Wellcare |
| Age | -0.019 | 0.043 | 0.051 | -0.023 | 0.008 |
| % Female | -0.029 | 0.048 | 0.024 | 0.007 | 0.023 |
| Household Size | 0.148 | -0.047 | -0.056 | -0.013 | 0.024 |
| % Hispanic/Latinx | 0.025 | -0.017 | -0.016 | 0.017 | -0.028 |
| % Black | -0.002 | -0.014 | -0.014 | 0.011 | -0.043 |
| % AAPI | -0.004 | -0.020 | -0.008 | -0.012 | -0.006 |
| % Native American | -0.001 | -0.003 | -0.004 | 0.006 | -0.006 |
| % White | 0.023 | 0.002 | -0.036 | -0.036 | 0.052 |
| % Unknown Race | -0.034 | 0.017 | 0.057 | 0.030 | -0.070 |
| % Primary Language Spanish | 0.019 | -0.027 | -0.029 | 0.026 | -0.016 |
| % Primary Language English | -0.019 | 0.027 | 0.028 | -0.025 | 0.021 |
| % 0-100% FPL | -0.018 | -0.021 | 0.005 | 0.001 | 0.011 |
| % 101-133% FPL | 0.020 | 0.015 | -0.002 | -0.003 | -0.010 |
| % 134-150% FPL | 0.009 | 0.005 | -0.004 | 0.002 | -0.001 |
| % 151-200% FPL | -0.007 | 0.012 | -0.004 | 0.006 | -0.002 |
| % 201-255% FPL | 0.003 | 0.008 | -0.003 | -0.007 | -0.004 |
| % 256-300% FPL | -0.015 | 0.004 | 0.002 | 0.004 | 0.003 |
| % 301-400% FPL | -0.008 | -0.003 | -0.002 | 0.003 | -0.002 |
| % over 401% FPL | -0.005 | -0.001 | -0.008 | -0.018 | 0.000 |
| % Veterans | -0.009 | 0.017 | 0.005 | 0.004 | -0.015 |
| % US Citizens | 0.026 | 0.029 | 0.008 | -0.003 | 0.003 |
| Doctors attached to Any MOUD Claim | 0.808 | -0.324 | 0.190 | 0.030 | -0.782 |
| Doctors Providing Bup with Any DEA Bup Waiver | 0.573 | -0.282 | 0.179 | 0.112 | -0.656 |
| Observations | 15839 | 47705 | 34786 | 39725 | 52134 |

Appendix Table 5: Cohen’s D - Second Calendar Year- Two Urban Counties

|  | (1) | (2) | (3) | (4) | (5) |
| --- | --- | --- | --- | --- | --- |
|  | Aetna | Anthem | Humana | Passport | Wellcare |
| Age | 0.011 | 0.035 | 0.017 | -0.022 | -0.029 |
| % Female | 0.014 | 0.019 | 0.007 | -0.019 | 0.007 |
| Household Size | 0.000 | -0.085 | -0.066 | 0.079 | -0.023 |
| % Hispanic/Latinx | 0.036 | -0.088 | -0.047 | 0.036 | 0.063 |
| % Black | -0.026 | -0.074 | -0.029 | 0.075 | -0.056 |
| % AAPI | 0.005 | -0.027 | -0.001 | 0.012 | 0.003 |
| % Native American | -0.017 | 0.016 | 0.024 | -0.010 | -0.052 |
| % White | -0.100 | 0.095 | -0.016 | -0.022 | -0.010 |
| % Unknown Race | 0.108 | 0.020 | 0.073 | -0.080 | 0.026 |
| % Primary Language Spanish | 0.040 | -0.112 | -0.055 | 0.047 | 0.062 |
| % Primary Language English | -0.039 | 0.111 | 0.054 | -0.046 | -0.063 |
| % 0-100% FPL | -0.011 | -0.019 | -0.016 | 0.009 | 0.038 |
| % 101-133% FPL | -0.019 | 0.011 | 0.022 | -0.004 | -0.030 |
| % 134-150% FPL | 0.034 | 0.011 | 0.008 | -0.012 | -0.027 |
| % 151-200% FPL | 0.034 | 0.013 | -0.023 | -0.003 | -0.008 |
| % 201-255% FPL | -0.009 | 0.004 | -0.014 | -0.012 | 0.012 |
| % 256-300% FPL | -0.020 | 0.017 | -0.029 | -0.006 | -0.023 |
| % 301-400% FPL | -0.014 | -0.054 | 0.015 | 0.013 | -0.054 |
| % over 401% FPL | 0.014 | -0.025 | 0.002 | -0.011 | -0.025 |
| % Veterans | 0.001 | 0.006 | 0.058 | -0.018 | -0.052 |
| % US Citizens | 0.060 | 0.124 | 0.080 | -0.101 | 0.009 |
| Doctors attached to Any MOUD Claim | 0.702 | -0.977 | 0.878 | -0.491 | 0.903 |
| Doctors Providing Bup with Any DEA Bup Waiver | 0.534 | -1.103 | 0.975 | -0.472 | 0.902 |
| Observations | 3519 | 8911 | 7146 | 15872 | 4851 |

Appendix Table 6: Cohen’s D - Second Calendar Year - All Other Counties

|  | (1) | (2) | (3) | (4) | (5) |
| --- | --- | --- | --- | --- | --- |
|  | Aetna | Anthem | Humana | Passport | Wellcare |
| Age | -0.020 | 0.037 | 0.038 | -0.031 | 0.008 |
| % Female | -0.016 | 0.047 | 0.014 | 0.005 | 0.023 |
| Household Size | 0.171 | -0.057 | -0.064 | -0.009 | 0.024 |
| % Hispanic/Latinx | 0.011 | -0.014 | -0.003 | 0.018 | -0.028 |
| % Black | -0.002 | -0.013 | -0.015 | 0.017 | -0.043 |
| % AAPI | 0.000 | -0.019 | -0.005 | -0.012 | -0.006 |
| % Native American | 0.003 | -0.008 | -0.004 | 0.008 | -0.006 |
| % White | 0.033 | -0.007 | -0.037 | -0.028 | 0.052 |
| % Unknown Race | -0.042 | 0.026 | 0.054 | 0.018 | -0.060 |
| % Primary Language Spanish | 0.021 | -0.027 | -0.014 | 0.022 | -0.016 |
| % Primary Language English | -0.022 | 0.028 | 0.015 | -0.020 | 0.021 |
| % 0-100% FPL | -0.026 | -0.012 | 0.009 | 0.006 | 0.011 |
| % 101-133% FPL | 0.029 | 0.006 | -0.004 | -0.008 | -0.010 |
| % 134-150% FPL | 0.007 | 0.006 | -0.007 | -0.000 | -0.001 |
| % 151-200% FPL | -0.012 | 0.009 | -0.008 | 0.007 | -0.002 |
| % 201-255% FPL | 0.010 | 0.008 | -0.003 | -0.014 | -0.004 |
| % 256-300% FPL | -0.013 | 0.003 | 0.003 | 0.003 | 0.003 |
| % 301-400% FPL | -0.007 | 0.002 | -0.005 | 0.007 | -0.002 |
| % over 401% FPL | -0.001 | -0.018 | -0.000 | -0.018 | 0.000 |
| % Veterans | -0.022 | 0.021 | 0.004 | 0.007 | -0.015 |
| % US Citizens | 0.027 | 0.028 | 0.000 | 0.002 | 0.003 |
| Doctors attached to Any MOUD Claim | 0.787 | -0.407 | 0.224 | 0.034 | -0.782 |
| Doctors Providing Bup with Any DEA Bup Waiver | 0.563 | -0.373 | 0.226 | 0.100 | -0.656 |
| Observations | 9998 | 30613 | 22377 | 26294 | 36555 |

Appendix Table 7: Plan Comprehensiveness and OUD Diagnosis and MOUD Receipt (Two Stage Residual Inclusion (Second Stage Logit) Model)

|  | First Calendar Year | | | |
| --- | --- | --- | --- | --- |
|  | OUD Dx (Uncontrolled) | | OUD Dx + MOUD (Uncontrolled) | |
|  | **Logit Reduced Form (1)** | **2SRI (2)** | **Logit Reduced Form (3)** | **2SRI (4)** |
| Outcome Variable | **OUD Dx** | **OUD Dx** | **OUD Dx + MOUD** | **OUD Dx + MOUD** |
| Assigned Plan Comprehensiveness (OR) | 1.098^***^ |  | 1.082^***^ |  |
|  | (0.0074) |  | (0.0113) |  |
| Realized Plan Comprehensiveness (OR) |  | 1.083^***^ |  | 1.055^***^ |
|  |  | (0.0073) |  | (0.0110) |
| *First Stage Instrument* |  |  |  |  |
| Assigned Plan Comprehensiveness |  | 0.926^***^ |  | 0.926^***^ |
|  |  | (0.00276) |  | (0.00276) |
| F-statistic for IV in first stage |  | 1833814 |  | 1833814 |
| N | 252791 | 252791 | 252791 | 252791 |
| Mean of Outcome Variable | 0.0370 | 0.0370 | 0.0152 | 0.0152 |
| SD of Outcome | 0.1887 | 0.1887 | 0.1225 | 0.1225 |
|  | Second Calendar Year | | | |
|  | OUD Dx (Uncontrolled) | | OUD Dx + MOUD (Uncontrolled) | |
|  | **Logit Reduced Form (1)** | **2SRI (2)** | **Logit Reduced Form (3)** | **2SRI (4)** |
| Outcome Variable | **OUD Dx** | **OUD Dx** | **OUD Dx + MOUD** | **OUD Dx + MOUD** |
| Assigned Plan Comprehensiveness (OR) | 1.099^***^ |  | 1.076^***^ |  |
|  | (0.0092) |  | (0.0099) |  |
| Realized Plan Comprehensiveness (OR) |  | 1.042^***^ |  | 1.074^***^ |
|  |  | (0.0088) |  | (0.0101) |
| *First Stage Instrument* |  |  |  |  |
| Assigned Plan Comprehensiveness |  | 0.729^***^ |  | 0.729^***^ |
|  |  | (0.02113) |  | (0.02113) |
| F-statistic for IV in first stage |  | 186232 |  | 186232 |
| N | 163808 | 163808 | 163808 | 163808 |
| Mean of Outcome Variable | 0.0582 | 0.0582 | 0.0340 | 0.0340 |
| SD of Outcome Variable | 0.2340 | 0.2340 | 0.1800 | 0.1800 |

Notes: Standard errors in parentheses. ^*^ *p* < 0.10, ^**^ *p* < 0.05, ^***^ *p* < 0.01. The Second Calendar Year has a smaller first-stage coefficient not because people are switching plans at the beginning of the second year but because plans on average are less generous the second year. (See Appendix Table 1 for 2^nd^ year Assigned and Realized stickiness rates). Covariates: % Black, % Hispanic/Latinx, % Missing/Other Race, % Female, % Age 18-33, % Age 34-49, % Age 50-64, Household Size.

Appendix Table 8: Full Version of Exhibit 2: Plan Comprehensiveness and OUD Diagnosis

|  | First Calendar Year | | | | | |
| --- | --- | --- | --- | --- | --- | --- |
|  | Uncontrolled | | County and Year FEs | | FEs and Covariates | |
|  | **OLS (1)** | **2SLS (2)** | **OLS (3)** | **2SLS (4)** | **OLS (5)** | **2SLS (6)** |
| Outcome Variable | **OUD Dx** | **OUD Dx** | **OUD Dx** | **OUD Dx** | **OUD Dx** | **OUD Dx** |
| Assigned Plan Comprehensiveness | 0.00286^***^ |  | 0.00325^***^ |  | 0.00325^***^ |  |
|  | (0.000242) |  | (0.000812) |  | (0.000951) |  |
| Realized Plan Comprehensiveness |  | 0.00306^***^ |  | 0.00350^***^ |  | 0.00356^***^ |
|  |  | (0.000258) |  | (0.000866) |  | (0.000930) |
| County and Year FEs |  |  | X | X | X | X |
| Covariates |  |  |  |  | X | X |
| *First Stage Instrument* |  |  |  |  |  |  |
| Assigned Plan Comprehensiveness |  | 0.938^***^ |  | 0.939^***^ |  | 0.939^***^ |
|  |  | (0.000693) |  | (0.00196) |  | (0.00192) |
| F-statistic for IV in first stage |  | 1833814 |  | 126450.8 |  | 125377.2 |
| N | 252,791 | 252,791 | 252,791 | 252,784 | 252,791 | 248,416 |
| Mean of Outcome Variable | 0.0370 | 0.0370 | 0.0370 | 0.0370 | 0.0370 | 0.0370 |
| SD of Outcome | 0.1887 | 0.1887 | 0.1887 | 0.1887 | 0.1887 | 0.1887 |
|  | Second Calendar Year | | | | | |
|  | Uncontrolled | | County and Year FEs | | FEs and Covariates | |
|  | **OLS (1)** | **2SLS (2)** | **OLS (3)** | **2SLS (4)** | **OLS (5)** | **2SLS (6)** |
| Outcome Variable | **OUD Dx** | **OUD Dx** | **OUD Dx** | **OUD Dx** | **OUD Dx** | **OUD Dx** |
| Assigned Plan Comprehensiveness | 0.00177^***^ |  | 0.00397^***^ |  | 0.00397^***^ |  |
|  | (0.000389) |  | (0.00122) |  | (0.00144) |  |
| Realized Plan Comprehensiveness |  | 0.00246^***^ |  | 0.00545^***^ |  | 0.00545^***^ |
|  |  | (0.000542) |  | (0.00153) |  | (0.00183) |
| County and Year FEs |  |  | X | X | X | X |
| Covariates |  |  |  |  | X | X |
| *First Stage Instrument* |  |  |  |  |  |  |
| Assigned Plan Comprehensiveness |  | 0.741^***^ |  | 0.717^***^ |  | 0.717^***^ |
|  |  | (0.00172) |  | (0.00908) |  | (0.00925) |
| F-statistic for IV in first stage |  | 186231.7 |  | 1045.583 |  | 1091.987 |
| N | 163,808 | 163,808 | 163,802 | 163,802 | 162,682 | 162,682 |
| Mean of Outcome Variable | 0.0582 | 0.0582 | 0.0582 | 0.0582 | 0.0582 | 0.0584 |
| SD of Outcome | 0.2340 | 0.2340 | 0.2340 | 0.2340 | 0.2340 | 0.2340 |

Notes: Standard errors in parentheses. ^*^ *p* < 0.10, ^**^ *p* < 0.05, ^***^ *p* < 0.01. The Second Calendar Year has a smaller first-stage coefficient not because people are switching plans at the beginning of the second year but because plans on average are less generous the second year. (See Appendix Table 1 for 2^nd^ year Assigned and Realized stickiness rates). Covariates: % Black, % Hispanic/Latinx, % Missing/Other Race, % Female, % Age 18-33, % Age 34-49, % Age 50-64, Household Size. See Appendix Table 7 for regression coefficients on covariates.

Appendix Table 9: Full Version of Exhibit 3: Plan Comprehensiveness and OUD Diagnosis + MOUD

|  | First Calendar Year | | | | | |
| --- | --- | --- | --- | --- | --- | --- |
|  | Uncontrolled | | County and Year FEs | | FEs and Covariates | |
|  | **OLS (1)** | **2SLS (2)** | **OLS (3)** | **2SLS (4)** | **OLS (5)** | **2SLS (6)** |
| Outcome Variable | **OUD Dx + MOUD** | **OUD Dx + MOUD** | **OUD Dx + MOUD** | **OUD Dx + MOUD** | **OUD Dx + MOUD** | **OUD Dx + MOUD** |
| Assigned Plan Comprehensiveness | 0.000817^***^ |  | 0.00120^***^ |  | 0.00124^***^ |  |
|  | (0.000157) |  | (0.000248) |  | (0.000281) |  |
| Realized Plan Comprehensiveness |  | 0.000872^***^ |  | 0.00130^***^ |  | 0.00136^***^ |
|  |  | (0.000166) |  | (0.000266) |  | (0.000276) |
| County and Year FEs |  |  | X | X | X | X |
| Covariates |  |  |  |  | X | X |
| *First Stage Instrument* |  |  |  |  |  |  |
| Assigned Plan Comprehensiveness |  | 0.938^***^ |  | 0.939^***^ |  | 0.939^***^ |
|  |  | (0.000693) |  | (0.00196) |  | (0.00192) |
| F-statistic for IV in first stage |  | 1833814 |  | 126450.8 |  | 125377.2 |
| N | 252,791 | 252,791 | 252,791 | 252,791 | 248,416 | 248,416 |
| Mean of Outcome Variable | 0.0152 | 0.0152 | 0.0152 | 0.0152 | 0.0154 | 0.0154 |
| SD of Outcome | 0.123 | 0.123 | 0.123 | 0.123 | 0.123 | 0.123 |
|  | Second Calendar Year | | | | | |
|  | Uncontrolled | | County and Year FEs | | FEs and Covariates | |
|  | **OLS (1)** | **2SLS (2)** | **OLS (3)** | **2SLS (4)** | **OLS (5)** | **2SLS (6)** |
| Outcome Variable | **OUD Dx + MOUD** | **OUD Dx + MOUD** | **OUD Dx + MOUD** | **OUD Dx + MOUD** | **OUD Dx + MOUD** | **OUD Dx + MOUD** |
| Assigned Plan Comprehensiveness | -0.0000574 |  | 0.00195^***^ |  | 0.00198^***^ |  |
|  | (0.000244) |  | (0.000355) |  | (0.000388) |  |
| Realized Plan Comprehensiveness |  | -0.0000799 |  | 0.00268^***^ |  | 0.00273^***^ |
|  |  | (0.000340) |  | (0.000464) |  | (0.000497) |
| County and Year FEs |  |  | X | X | X | X |
| Covariates |  |  |  |  | X | X |
| *First Stage Instrument* |  |  |  |  |  |  |
| Assigned Plan Comprehensiveness |  | 0.741^***^ |  | 0.717^***^ |  | 0.717^***^ |
|  |  | (0.00172) |  | (0.00908) |  | (0.00925) |
| F-statistic for IV in first stage |  | 186231.7 |  | 1045.583 |  | 1116.475 |
| N | 163,808 | 163,808 | 163,808 | 163,808 | 162,682 | 162,682 |
| Mean of Outcome Variable | 0.0340 | 0.0340 | 0.0340 | 0.0340 | 0.0340 | 0.0340 |
| SD of Outcome | 0.181 | 0.181 | 0.181 | 0.181 | 0.181 | 0.181 |

Notes: Standard errors in parentheses. ^*^ *p* < 0.10, ^**^ *p* < 0.05, ^***^ *p* < 0.01. The Second Calendar Year has a smaller first-stage coefficient not because people are switching plans at the beginning of the second year but because plans on average are less generous the second year. (See Appendix Table 1 for 2^nd^ year Assigned and Realized stickiness rates). Covariates: % Black, % Hispanic/Latinx, % Missing/Other Race, % Female, % Age 18-33, % Age 34-49, % Age 50-64, Household Size. See Appendix Table 7 for regression coefficients on covariates.

Appendix Table 10 Subpopulation Analysis - Males 25-34

|  | First Calendar Year | | Second Calendar Year | |
| --- | --- | --- | --- | --- |
|  | **2SLS (1)** | **2SLS (2)** | **2SLS (3)** | **2SLS (4)** |
| Outcome Variable | **OUD Dx** | **OUD Dx + MOUD** | **OUD Dx** | **OUD Dx + MOUD** |
| Realized Plan Comprehensiveness | 0.000932 | 0.0000297 | -0.0000769 | 0.000641 |
|  | (0.000870) | (0.000187) | (0.00149) | (0.000801) |
| Male24_35*Realized Plan Comprehensiveness (Interaction) | 0.0115^***^ | 0.00422^***^ | 0.0168^***^ | 0.00977^***^ |
|  | (0.000976) | (0.000589) | (0.00137) | (0.00114) |
| County and Year FEs | X | X | X | X |
| *First Stage Instrument (Realized)* |  |  |  |  |
| Assigned Plan Comprehensiveness | 0.926^***^ | 0.926^***^ | 0.728^***^ | 0.728^***^ |
|  | (0.00287) | (0.00287) | (0.0214) | (0.0214) |
| Male24_35*Assigned Plan Comprehensiveness (Interaction) | 0.00552^***^ | 0.00552^***^ | 0.00386 | 0.00386 |
|  | (0.00169) | (0.00169) | (0.00321) | (0.00321) |
| *First Stage Instrument (Realized Interaction)* |  |  |  |  |
| Assigned Plan Comprehensiveness | -0.00810^***^ | -0.00810^***^ | -0.0441^***^ | -0.0441^***^ |
|  | (0.000513) | (0.000513) | (0.00195) | (0.00195) |
| Male24_35*Assigned Plan Comprehensiveness (Interaction) | 0.989^***^ | 0.989^***^ | 0.985^***^ | 0.985^***^ |
|  | (0.00308) | (0.00308) | (0.0126) | (0.0126) |
| F-statistic for IV in first stage | 55,582.18 | 55,582.18 | 1,156.08 | 1,156.08 |
| N | 252,784 | 252,784 | 162,682 | 162,682 |
| Mean of Outcome Variable | 0.0370 | 0.0155 | 0.0586 | 0.0342 |
| SD of Outcome | 0.190 | 0.124 | 0.235 | 0.182 |

Note: Standard errors in parentheses; SEs clustered at the county level. ^*^*p* < 0.10, ^**^ *p* < 0.05, ^***^ *p* < 0.01. For the estimation of these models, we use the sample of beneficiaries and interact the plan comprehensiveness variable with a dummy variable for those individuals in the subpopulation of interest.

Appendix Table 11: Subpopulation Analysis – Two Largest Urban Counties

|  | First Calendar Year | | | | | |
| --- | --- | --- | --- | --- | --- | --- |
|  | **2SLS** | | | | | |
| Outcome Variable | **OUD Dx** | | | **OUD Dx + MOUD** | | |
| Model | Main Spec. | Rural Counties Only | Urban Counties Only | Main Spec. | Rural Counties Only | Urban Counties Only |
| Realized Plan Comprehensiveness | 0.00350^***^ | 0.00245^***^ | 0.00602^***^ | 0.00120^***^ | 0.00116^***^ | 0.00159^***^ |
|  | (0.000866) | (0.000604) | (0.000729) | (0.000248) | (0.000337) | (0.000196) |
| County and Year FEs | X | X | X | X | X | X |
| *First Stage Instrument* |  |  |  |  |  |  |
| Assigned Plan Comprehensiveness | 0.939^***^ | 0.925^***^ | 0.935^***^ | 0.939^***^ | 0.925^***^ | 0.935^***^ |
|  | (0.00196) | (0.00280) | (0.00250) | (0.00196) | (0.00280) | (0.00250) |
| F-statistic for IV in first stage | 126450.8 | 109063.4 | 140250.8 | 126450.8 | 109063.4 | 140250.8 |
| N | 252791 | 190182 | 62602 | 252791 | 190182 | 62602 |
| Mean of Outcome Variable | 0.037 | 0.037 | 0.037 | 0.0154 | 0.017 | 0.011 |
| SD of Outcome | 0.189 | 0.189 | 0.189 | 0.123 | 0.189 | 0.102 |
|  | Second Calendar Year | | | | | |
|  | **2SLS** | | | | | |
| Outcome Variable | **OUD Dx** | | | **OUD Dx + MOUD** | | |
| Model | Main Spec. | Rural Counties Only | Urban Counties Only | Main Spec. | Rural Counties Only | Urban Counties Only |
| Realized Plan Comprehensiveness | 0.00545^***^ | 0.00368^***^ | 0.00930^***^ | 0.00348^***^ | 0.00332^***^ | 0.00376^***^ |
|  | (0.00153) | (0.00113) | (0.00153) | (0.000609) | (0.000867) | (0.000116) |
| County and Year FEs | X | X | X | X | X | X |
| *First Stage Instrument* |  |  |  |  |  |  |
| Assigned Plan Comprehensiveness | 0.717^***^ | 0.694^***^ | 0.816^***^ | 0.717^***^ | 0.694^***^ | 0.816^***^ |
|  | (0.00908) | (0.00905) | (0.00189) | (0.00908) | (0.00905) | (0.00189) |
| F-statistic for IV in first stage | 1045.583 | 5867.316 | 186600.5 | 1045.583 | 5867.316 | 186600.5 |
| N | 163808 | 123980 | 39822 | 163808 | 123980 | 39822 |
| Mean of Outcome Variable | 0.058 | 0.060 | 0.0534 | 0.034 | 0.037 | 0.025 |
| SD of Outcome | 0.2340 | 0.237 | 0.225 | 0.181 | 0.188 | 0.157 |

Note: Standard errors in parentheses; SEs clustered at the county level. ^*^*p* < 0.10, ^**^ *p* < 0.05, ^***^ *p* < 0.01. For the estimation of these models, we use the sample of beneficiaries and interact the plan comprehensiveness variable with a dummy variable for those individuals in the subpopulation of interest.

Appendix Table 12: Plan Comprehensiveness and OUD Diagnosis and MOUD Receipt (Robustness Excluding Injectable Naltrexone)

|  | First Calendar Year | | | |
| --- | --- | --- | --- | --- |
|  | OUD Dx | | OUD Dx + MOUD | |
|  | **Reduced Form (1)** | **2SLS (2)** | **Reduced Form (3)** | **2SLS (4)** |
| Outcome Variable | **OUD Dx** | **OUD Dx** | **OUD Dx + MOUD** | **OUD Dx + MOUD** |
| Assigned Plan Comprehensiveness (No Inj. Naltrexone) | 0.00356^***^ |  | 0.000994^***^ |  |
|  | (0.00103) |  | (0.000327) |  |
| Realized Plan Comprehensiveness (No Inj. Naltrexone) |  | 0.00325^***^ |  | 0.000658^***^ |
|  |  | (0.00100) |  | (0.000222) |
| County and Year FEs | X | X | X | X |
| *First Stage Instrument* |  |  |  |  |
| Assigned Plan Comprehensiveness |  | 0.929^***^ |  | 0.929^***^ |
|  |  | (0.00261) |  | (0.00261) |
| F-statistic for IV in first stage |  | 126410.6 |  | 126410.6 |
| N | 252749 | 252749 | 252749 | 252749 |
| Mean of Outcome Variable | 0.0369 | 0.0369 | 0.0152 | 0.0152 |
| SD of Outcome | 0.1887 | 0.1887 | 0.1225 | 0.1225 |
|  | Second Calendar Year | | | |
|  | OUD Dx | | OUD Dx + MOUD | |
|  | **Reduced Form (1)** | **2SLS (2)** | **Reduced Form (3)** | **2SLS (4)** |
| Outcome Variable | **OUD Dx** | **OUD Dx** | **OUD Dx + MOUD** | **OUD Dx + MOUD** |
| Assigned Plan Comprehensiveness (No Inj. Naltrexone) | 0.00318^*^ |  | 0.00147^**^ |  |
|  | (0.00161) |  | (0.000637) |  |
| Realized Plan Comprehensiveness (No Inj. Naltrexone) |  | 0.00146 |  | 0.00187^*^ |
|  |  | (0.00222) |  | (0.000956) |
| County and Year FEs | X | X | X | X |
| *First Stage Instrument* |  |  |  |  |
| Assigned Plan Comprehensiveness |  | 0.642^***^ |  | 0.642^***^ |
|  |  | (0.0200) |  | (0.0200) |
| F-statistic for IV in first stage |  | 1033.9 |  | 1033.9 |
| N | 163781 | 163781 | 163781 | 163781 |
| Mean of Outcome Variable | 0.0581 | 0.0581 | 0.0337 | 0.0337 |
| SD of Outcome | 0.2340 | 0.2340 | 0.1800 | 0.1800 |

Notes: Standard errors in parentheses. ^*^ *p* < 0.10, ^**^ *p* < 0.05, ^***^ *p* < 0.01. The Second Calendar Year has a smaller first-stage coefficient not because people are switching plans at the beginning of the second year but because plans on average are less generous the second year. (See Appendix Table 1 for 2^nd^ year Assigned and Realized stickiness rates). Covariates: % Black, % Hispanic/Latinx, % Missing/Other Race, % Female, % Age 18-33, % Age 34-49, % Age 50-64, Household Size.

Appendix Table 13: Service Comprehensiveness and OUD Diagnosis and MOUD Receipt (Robustness Excluding Buprenorphine and Injectable Naltrexone)

|  | First Calendar Year | | | |
| --- | --- | --- | --- | --- |
|  | OUD Dx | | OUD Dx + MOUD | |
|  | **Reduced Form (1)** | **2SLS (2)** | **Reduced Form (3)** | **2SLS (4)** |
| Outcome Variable | **OUD Dx** | **OUD Dx** | **OUD Dx + MOUD** | **OUD Dx + MOUD** |
| Assigned Service Comprehensiveness | 0.00358^***^ |  | 0.000991^***^ |  |
|  | (0.00103) |  | (0.000332) |  |
| Realized Service Comprehensiveness |  | 0.00348^***^ |  | 0.000662^***^ |
|  |  | (0.00105) |  | (0.000235) |
| County and Year FEs | X | X | X | X |
| *First Stage Instrument* |  |  |  |  |
| Assigned Service Comprehensiveness |  | 0.909^***^ |  | 0.909^***^ |
|  |  | (0.00248) |  | (0.00248) |
| F-statistic for IV in first stage |  | 134002.9 |  | 134002.9 |
| N | 252749 | 252749 | 252749 | 252749 |
| Mean of Outcome Variable | 0.0369 | 0.0369 | 0.0152 | 0.0152 |
| SD of Outcome | 0.1887 | 0.1887 | 0.1225 | 0.1225 |
|  | Second Calendar Year | | | |
|  | OUD Dx | | OUD Dx + MOUD | |
|  | **Reduced Form (1)** | **2SLS (2)** | **Reduced Form (3)** | **2SLS (4)** |
| Outcome Variable | **OUD Dx** | **OUD Dx** | **OUD Dx + MOUD** | **OUD Dx + MOUD** |
| Assigned Service Comprehensiveness | 0.00584^***^ |  | 0.00176^**^ |  |
|  | (0.00130) |  | (0.000783) |  |
| Realized Service Comprehensiveness |  | 0.00524^***^ |  | 0.00166^***^ |
|  |  | (0.00131) |  | (0.000605) |
| County and Year FEs | X | X | X | X |
| *First Stage Instrument* |  |  |  |  |
| Assigned Service Comprehensiveness |  | 0.911^***^ |  | 0.911^***^ |
|  |  | (0.00320) |  | (0.00320) |
| F-statistic for IV in first stage |  | 80963.8 |  | 80963.8 |
| N | 163781 | 163781 | 163781 | 163781 |
| Mean of Outcome Variable | 0.0581 | 0.0581 | 0.0337 | 0.0337 |
| SD of Outcome | 0.2340 | 0.2340 | 0.1800 | 0.1800 |

Notes: Standard errors in parentheses. ^*^ *p* < 0.10, ^**^ *p* < 0.05, ^***^ *p* < 0.01. The Second Calendar Year has a smaller first-stage coefficient not because people are switching plans at the beginning of the second year but because plans on average are less generous the second year. (See Appendix Table 1 for 2^nd^ year Assigned and Realized stickiness rates). Covariates: % Black, % Hispanic/Latinx, % Missing/Other Race, % Female, % Age 18-33, % Age 34-49, % Age 50-64, Household Size.

We identified MOUD prescribers using National Provider Identifiers (NPI) in the Medicaid RX files. We used the CMS National Plan and Provider Enumeration System (NPPES) to connect NPIs in the RX file to provider names and zip codes, which allowed us to merge Drug Enforcement Administration (DEA) buprenorphine waiver data to the Medicaid claims,[^24,25^](https://paperpile.com/c/yJTsvW/M7MZk+zZBy5) to identify providers with buprenorphine waivers. Data on DEA waivers were taken from DEA Active Controlled Substances Act (CSA) Registrants data purchased through the National Technical Information Service (NTIS).^8^

Appendix Table 14: IV Covariate Models, with Provider Variable 1 (Raw number of Unique MOUD Providers)

|  | First Calendar Year | | Second Calendar Year | |
| --- | --- | --- | --- | --- |
|  | **2SLS (1)** | **2SLS (2)** | **2SLS (3)** | **2SLS (4)** |
| Outcome Variable | **OUD Dx** | **OUD Dx + MOUD** | **OUD Dx** | **OUD Dx + MOUD** |
| Realized Plan Comprehensiveness | 0.00371^***^ | 0.00147^***^ | 0.00616^***^ | 0.00418^***^ |
|  | (0.000922) | (0.000279) | (0.00156) | (0.000650) |
| *Covariates* |  |  |  |  |
| Doctors attached to Any MOUD Claim | 0.0717^***^ | 0.0630^***^ | 0.139^***^ | 0.139^***^ |
|  | (0.0139) | (0.0111) | (0.0211) | (0.0211) |
| Female (Ref: Male) | -0.0235^***^ | -0.00837^***^ | -0.0330^***^ | -0.0171^***^ |
|  | (0.00190) | (0.00110) | (0.00285) | (0.00220) |
| Household Size (1-7+) | -0.0118^***^ | -0.00371^***^ | -0.0168^***^ | -0.00816^***^ |
|  | (0.000527) | (0.000384) | (0.000766) | (0.000664) |
| Hispanic/Latinx (Ref: White) | -0.0361^***^ | -0.0137^***^ | -0.0559^***^ | -0.0320^***^ |
|  | (0.00571) | (0.00118) | (0.00770) | (0.00339) |
| Black (Ref: White) | -0.0274^***^ | -0.0129^***^ | -0.0473^***^ | -0.0311^***^ |
|  | (0.00266) | (0.00194) | (0.00325) | (0.00179) |
| Race Unknown/Missing (Ref: White) | -0.0247^***^ | -0.0108^***^ | -0.0372^***^ | -0.0224^***^ |
|  | (0.00263) | (0.00107) | (0.00358) | (0.00173) |
| Age 18-33 (Ref: 50-64) | 0.0277^***^ | 0.0118^***^ | 0.0420^***^ | 0.0266^***^ |
|  | (0.00189) | (0.00125) | (0.00260) | (0.00207) |
| Age 34-49 (Ref: 50-64) | 0.0375^***^ | 0.0173^***^ | 0.0583^***^ | 0.0376^***^ |
|  | (0.00202) | (0.00200) | (0.00386) | (0.00386) |
| County and Year FEs | X | X | X | X |
| *First Stage Instrument* |  |  |  |  |
| Assigned Plan Comprehensiveness | 0.925^***^ | 0.925^***^ | 0.727^***^ | 0.727^***^ |
|  | (0.00283) | (0.00283) | (0.0208) | (0.0208) |
| F-statistic for IV in first stage | 118165.5 | 118165.5 | 1019.751 | 1019.751 |
| N | 247759 | 252791 | 162682 | 162682 |
| Mean of Outcome Variable | 0.0372 | 0.0155 | 0.0584 | 0.0340 |
| SD of Outcome | 0.189 | 0.123 | 0.234 | 0.181 |

Note: Standard errors in parentheses; SEs clustered at the county level. ^*^*p* < 0.10, ^**^ *p* < 0.05, ^***^ *p* < 0.01

Appendix Table 15: IV Covariate Models, with Provider Variable 2 (Unique Buprenorphine Providers with DEA Waivers)

|  | First Calendar Year | | Second Calendar Year | |
| --- | --- | --- | --- | --- |
|  | **2SLS (1)** | **2SLS (2)** | **2SLS (3)** | **2SLS (4)** |
| Outcome Variable | **OUD Dx** | **OUD Dx + MOUD** | **OUD Dx** | **OUD Dx + MOUD** |
| Realized Plan Comprehensiveness | 0.00360^***^ | 0.00139^***^ | 0.00591^***^ | 0.00394^***^ |
|  | (0.000939) | (0.000282) | (0.00161) | (0.000633) |
| *Covariates* |  |  |  |  |
| Doctors Providing Bup with Any DEA Waiver | 0.119^***^ | 0.123^***^ | 0.226^***^ | 0.244^***^ |
|  | (0.0250) | (0.0222) | (0.0395) | (0.0386) |
| Female (Ref: Male) | -0.0235^***^ | -0.00841^***^ | -0.0330^***^ | -0.0171^***^ |
|  | (0.00191) | (0.00111) | (0.00288) | (0.00223) |
| Household Size (1-7+) | -0.0119^***^ | -0.00371^***^ | -0.0168^***^ | -0.00817^***^ |
|  | (0.000525) | (0.000386) | (0.000765) | (0.000665) |
| Hispanic/Latinx (Ref: White) | -0.0362^***^ | -0.0138^***^ | -0.0561^***^ | -0.0322^***^ |
|  | (0.00571) | (0.00118) | (0.00768) | (0.00336) |
| Black (Ref: White) | -0.0274^***^ | -0.0130^***^ | -0.0473^***^ | -0.0311^***^ |
|  | (0.00266) | (0.00195) | (0.00326) | (0.00179) |
| Race Unknown/Missing (Ref: White) | -0.0249^***^ | -0.0109^***^ | -0.0374^***^ | -0.0225^***^ |
|  | (0.00261) | (0.00107) | (0.00356) | (0.00172) |
| Age 18-33 (Ref: 50-64) | 0.0278^***^ | 0.0119^***^ | 0.0420^***^ | 0.0266^***^ |
|  | (0.00190) | (0.00126) | (0.00261) | (0.00208) |
| Age 34-49 (Ref: 50-64) | 0.0377^***^ | 0.0174^***^ | 0.0584^***^ | 0.0378^***^ |
|  | (0.00204) | (0.00203) | (0.00389) | (0.00390) |
| County and Year FEs | X | X | X | X |
| *First Stage Instrument* |  |  |  |  |
| Assigned Plan Comprehensiveness | 0.926^***^ | 0.926^***^ | 0.729^***^ | 0.729^***^ |
|  | (0.00281) | (0.00281) | (0.0205) | (0.0205) |
| F-statistic for IV in first stage | 121040 | 121040 | 1058.169 | 1058.169 |
| N | 246112 | 246112 | 162682 | 162682 |
| Mean of Outcome Variable | 0.0373 | 0.0155 | 0.0586 | 0.0342 |
| SD of Outcome | 0.190 | 0.124 | 0.235 | 0.182 |

Note: Standard errors in parentheses; SEs clustered at the county level. ^*^*p* < 0.10, ^**^ *p* < 0.05, ^***^ *p* < 0.01

Appendix Table 16: TAR Checklist - Medicaid MCO Service Coverage and Diagnosis and Treatment of Opioid Use Disorder: Evidence from Quasi-Random Auto-Assignment in Kentucky

| **Category** | **Description** |
| --- | --- |
| **Data** |  |
| Files, Years, and Release Versions | Data from this study came from the TAF Other Services, TAF Inpatient, TAF Demographics and Eligibility, TAF Prescription Claims, and Annual Provider Files for 2016 (Release 2), 2017 (Release 2), and 2018 (Release 2). This study uses a 100% TAF sample of Medicaid beneficiaries aged 18-64, in Kentucky. |
| **Cohort** |  |
| Eligibility Criteria | We limited our analysis to individuals newly enrolled in Medicaid managed care plans, identified through new beneficiary IDs in the DE file each year. In Kentucky, 100% of Medicaid enrollees are provided care through managed care plans. |
| Enrollment Span | We limited our analysis to individuals with at least three months of continuous enrollment per calendar year, defined as a break of no more than one month at any point during enrollment. |
| Scope of Benefits | Our sample was limited to individuals with full scope or comprehensive benefits based on their benefit status for the plurality of months they were enrolled in Medicaid in the year. |
| Dual Eligibility | We excluded any individual with dual eligibility for both Medicaid and Medicare in any month of the year from our analysis. We excluded those with partial or full dual eligibility based on the monthly eligibility status observed latest in the year. Individuals with unknown dual eligibility status were not excluded. |
| **State and Territory Exclusions** |  |
| Criteria | This study exclusively focuses on Kentucky. All other states were excluded. |
| State variation table | Appendix Section 1 includes additional tables on key variables of interest, enrollment numbers, and assigned and realized managed care plans. |
| **Special Considerations** |  |
| Encounter Data | Enrollees participating in comprehensive managed care plans for more than three months in the calendar year were included in the analysis. |
| Spending | Not applicable to this study. |
| Using TAF with Predecessor Medicaid Analytic eXtract (MAX) Data | This study includes TAF data only (we do not include any years with MAX data). |

Citation: T-MSIS Analytic Files Analysis Reporting (TAR) Checklist. Medicaid Data Learning Network; 2024.
